# Experience of initiating collaboration of traditional healers in managing HIV and AIDS in Tanzania

**DOI:** 10.1186/1746-4269-3-6

**Published:** 2007-01-26

**Authors:** Edmund J Kayombo, Febronia C Uiso, Zakaria H Mbwambo, Rogasian L Mahunnah, Mainen J Moshi, Yasin H Mgonda

**Affiliations:** 1 Institute of Traditional Medicine, Muhimbili University College of health Sciences, P. O. Box 65001, Dar-es-Salaam, Tanzania; 2 School of Medicine, Department of Internal Medicine, Muhimbili University College of health Sciences, P. O. Box 65001, Dar-es-Salaam, Tanzania

## Abstract

Collaboration between traditional healers and biomedical practitioners is now being accepted by many African countries south of the Sahara because of the increasing problem of HIV/AIDS. The key problem, however, is how to initiate collaboration between two health systems which differ in theory of disease causation and management. This paper presents findings on experience learned by initiation of collaboration between traditional healers and the Institute of Traditional Medicine in Arusha and Dar-es-Salaam Municipalities, Tanzania where 132 and 60 traditional healers respectively were interviewed. Of these 110 traditional healers claimed to be treating HIV/AIDS. The objective of the study was to initiate sustainable collaboration with traditional healers in managing HIV/AIDS. Consultative meetings with leaders of traditional healers' associations and government officials were held, followed by surveys at respective traditional healers' "vilinge" (traditional clinics). The findings were analysed using both qualitative and quantitative methods. The findings showed that influential people and leaders of traditional healers' association appeared to be gatekeepers to access potential good healers in the two study areas. After consultative meetings these leaders showed to be willing to collaborate; and opened doors to other traditional healers, who too were willing to collaborate with the Institute of Traditional Medicine in managing HIV/AIDS patients. Seventy five percent of traditional healers who claimed to be treating HIV/AIDS knew some HIV/AIDS symptoms; and some traditional healers attempted to manage these symptoms. Even though, they were willing to collaborate with the Institute of Traditional Medicine there were nevertheless some reservations based on questions surrounding sharing from collaboration. The reality of past experiences of mistreatment of traditional healers in the colonial period informed these reservations. General findings suggest that initiating collaboration is not as easy as it appears to be from the literature, if it is to be meaningful; and thus we are calling for appropriate strategies to access potential healers targeted for any study designed with sustainability in mind.

## Background

*'As such, if we continue to encourage them and work with them, I see traditional healers as partners in the development of vaccines and treatments for AID*S' Nguma 2005 p 3, UN Office for the Coordination of Humanitarian Affairs.

Since the beginning of the AIDS epidemic, patients have consulted both biomedical doctors and traditional healers for all kinds of physical, emotional and spiritual ills [1-5]. Attempts to bring biomedical and traditional healthcare together to people living with HIV/AIDS have been made, at least since the early 1990s, [2,3,5,6], when WHO recommended that traditional medicine be included in national responses to HIV; and hence the idea of collaboration [1,2,6].

To handle the HIV/AIDS epidemic requires a mobilization of existing and potential resources of two health systems for coordinated controlled effort from the grassroots to national level [3,5,7]. Thus the need for the collaboration of two healthcare systems is now more important in Sub-saharan Africa due to the ever increasing burden of HIV/AIDS [1,5,8-10]. It has been noted that in behaviour for seeking healthcare support, a patient may start with allopathic medicine; and then move to traditional medicine and vice versa depending on what has been perceived to be the cause of disease/illness [3,4]. Nevertheless, the problem is that each health system works independently because they have different theories on causation of disease/illness and its management [3,10,11]; even though all are focusing on patients' welfare. It is being acknowledged that, healthcare systems are socially and culturally constructed in any community; these constructions include beliefs about the causes of illness and norms governing choice and evaluation of treatment as well as power relations [2,10,12], therefore the socio-cultural aspects need to be taken into account in any healthcare system, and especially when fighting against the spread of HIV/AIDS.

In the Regional Workshop [13] African governments expressed a need for a concerted, systematic and sustained effort at both local and regional levels to support and validate African traditional medicine on several fronts including evaluation of traditional remedies, spiritual aspects of healing, HIV prevention and care, standardization of processing and packaging traditional remedies, as well as protection of indigenous knowledge and intellectual property rights [13]. It is vital that all methods of healthcare delivery and prevention are explored in order to curb this epidemic [10,13].

As a result of this Regional Workshop [13] and also UNAIDS [1] recommendations, several models of collaboration between biomedical and traditional health practitioners have been developed like that of Traditional Healers and Modern Practitioners Together Against AIDS (THETA) in Uganda, and Tanga AIDS Working Group (TAWG) in Tanzania and Zimbabwe National Traditional Healers Association (ZINATHA) in Zimbabwe. The groups and associations are examples where biomedical healthcare practitioners are collaborating with traditional healthcare practitioners [1,3,13]. Not only are such collaborations happening in these countries but also in Mozambique, South Africa and Cameroon to mention a few; attempts are being made to collaborate with traditional healers on the management of HIV/AIDS [1,7].

The idea is sound, but the problem relates to how collaboration is being initiated and carried out when biomedicine and traditional medicine differ in theories on causation of disease/illness and even in management of the health problem in question [7,13]. For example, in traditional medicine, the key issues include the following; firstly, ill health and other forms of misfortune are believed to be caused by either social causes (witchcraft, sorcery or evil eye), or by supernatural causes (gods, spirits, ancestral spirits) or natural causes (accidents, weather, living environment, heritage, etc) [14,15]. Secondly, divination is one of the main tools in identifying both illnesses/diseases and causes [4]. Diviners, skilled in the process of divination, and supplemented with skilled history taking during interviews with patients/relatives can vividly see indicators for the type of illnesses/diseases and their causes [4]. Thirdly, success in treating patient's illness/disease is measured by pragmatic results i. e. relieves suffering and allows harmonious living in the community [15-17].

Nevertheless, traditional healers (practitioners of traditional medicine) have scanty information about AIDS because it is a new disease, and they lack standardized training [3,13,18] in healthcare. In addition, herbal treatments have often never been rigorously evaluated, nor are they always properly prepared or standardized, are usually poorly packaged and preserved, limiting their usefulness and accessibility to the immediate production site [2,13].

In biomedicine, on the other hand, key issues are; firstly disease is seen as deviation from normal values, accompanied by abnormalities in structure or function by body organs or systems. Secondly, pathological processes are firmly identified by blood tests, X rays, scans and other investigations usually carried out in specialised laboratories or clinics. Thirdly, the broad model of biomedicine is mainly directed towards discovering and quantifying physico-chemical information about the patient, rather than less measurable societal and emotional factors which are common in the community where the patient lives. The ideology instilled in many biomedical health workers has in turn led them to be prejudiced against traditional medicine and traditional healers [7,10]. Further more, the impact of biomedical ideology of disease causation has led national governments and ministries of health to be less enthusiastic about co-operation with traditional healers [12]. The question which we are asking when thinking about collaboration is: 'Have biomedical practitioners now changed their views on traditional medicine since the rise in incidence of HIV/AIDS?'

Documented systematic attempts at collaboration with the traditional health care sector have mainly involved organising traditional healers into national organisations, training programmes for healers, and laboratory testing of traditionally used herbs [2,12,19]. Achievements in these programmes included willingness of hospitals and clinics in some countries, such as Kenya and Tanzania, to provide on-site practice facilities to traditional healers, combined with training programmes [20,21]. But considerable challenges remain. For example, the process of actualizing collaboration is not transparent meaning it is possible for it to be dominated by one group (often biomedical practitioners) in the so-called 'collaboration process'. The sustainability of such collaboration is highly questionable. Any effective collaboration requires a mutual understanding through dialogue, an exchange of materials and technology and signing memorandum of understanding (MoU). MoU in this article is defined as freely negotiated agreement between parties in a collaborative undertaking. MoU contains intentions, obligations and mutual responsibilities between the collaborating parties. The role of MoU is, therefore, to bring into line functions and create more avenues for collaboration for the mutual benefits of each party. Furthermore, the question of who will receive which benefits from the collaboration remains.

### Framework for collaboration with traditional healers

Collaboration, literally, consists of working together with one or more others on mutual understanding which may accompany signing a memorandum of understanding. Collaboration between traditional healers and biomedical practitioners in African countries south of the Sahara is ever more important now in improving healthcare because it is likely to widen the scope of sharing and collecting information and, allows for shared leadership, decisions, ownership, vision, and responsibility [1,2,4,21] in the management of health problems and especially HIV/AIDS.

The proposed framework for collaboration between traditional healers and biomedical practitioners assumes that as an individual, group or community discovers health care solutions they also expand capacity within collaboration. Further when creativity, skills, self-sufficiency, and motivation, accompany collaboration, possibilities are extended, doors are opened, capacity expanded, and success realized in improving healthcare to the community.

However, this can only be achieved if some of the key elements proposed by King [2] are included during the initiation of a collaboration program. These key elements are; building mutual respect between biomedical and traditional health practitioners through dialogue on matters of interest, signing a memorandum of understanding; stressing complementarities of both systems by referral from one health system to another. Other proposed key elements to cement collaboration are showing humility and respect during workshop regardless of level of education; cultivating transparency through dialogue and negotiation, eagerness to learn from one another. Consistent exchange of information on management of illnesses/diseases, materials and technology used in preparation and dispensing are also key to collaboration. But most importantly is the selection of genuine healers i. e recognised traditional healers in the community through competence in managing diseases/illnesses and trustworthiness.

Furthermore, involving community leaders and members in selecting traditional healers for collaboration by rating the traditional healers on the level of competence in managing claimed diseases/illnesses they are treating and trustworthiness on the basis of clients' views. This can be complemented by involving biomedical health workers listing traditional healers on the basis of informal referral cases noted in their respective catchments areas. All in all, there is a need of planning for a long-term collaboration which can be measured by willingness to participate on key issues like vaccination and prevention of infectious diseases. The number of collaboration meetings and number of attendances can also be used as a measure of collaboration.

In addition, discussing differences and conflicts in cosmologies, evolution and changes in both health systems can be assessed through openness in dialogue and exchange of information on issues. Forming a dedicated and caring team which can manifest itself through willingness in participation on how to care for patients and looking for best options to arrest the illness and prevention is another avenue for assessing the level of collaboration. Collaborating with local institutions in enhancing and acknowledging practices within healthcare; opening/running or advocating collaborative clinics can be measured by the number of collaborating clinics opened in an area; including herbal research and/or provision of herbal medicine through joint research on specific illnesses/diseases. This collaboration can further be consolidated by adopting a comprehensive training approach focussing on key areas of prevention and treatment including a strong monitoring and evaluation component [2] with jointly accepted parameters.

The research team of this study critically scrutinized the key elements cited by King [2] and attempted to operationalise them in a fieldwork situation. This article, thus, presents the experience learned by initiating collaboration between Institute of Traditional Medicine (ITM) of Muhimbili University College (with biomedical practitioners) and traditional healers using the proposed framework for collaboration in Arusha and Dar-es-Salaam Municipalities specifically focused on:-

- the entry point to access traditional healers

- Knowledge on HIV/AIDS

- Handling cultural aspect like rituals in healthcare

- What traditional healers want from the collaboration

### Research methodology

The study was carried out in Dar-es-Salaam and Arusha Municipalities, Tanzania from July to November in 2003. The two areas were purposefully chosen because firstly, they have many traditional healers who claimed to be treating HIV/AIDS patients. Secondly, they were among the areas with high prevalence of HIV/AIDS when compared to other urban centres in Tanzania [22]. Thirdly, due to their proximity to Institute of Traditional Medicine for the cases in the study which need laboratory work.

Arusha Municipality is in the northern part of Tanzania and also the head quarter of the Arusha region. It has a total population of 282,712, and of these 143,675 are females [23]. The indigenous people in the region are the Wamasai, Wameru, Wairaqw and Waarusha ethnic groups. The main occupations of these ethnic groups are herding and farming. In recent years Arusha Municipality is receiving many migrants from all over the country because of the increasing number of manufacturing industries and mining. Also Arusha hosts international institutions such as UN-ICTR and an East African Community. With the increasing number of migrants and workers from international institutions there is a lot of social interaction which sometimes leads to HIV infection. The prevalence of HIV/AIDS infection in the Arusha Municipality is estimated to be around 11% [22].

Dar-es-Salaam city on the other hand is on the eastern coast of Tanzania. Dar-es-Salaam is the regional headquarter and former capital of the country (now it is Dodoma). It is the centre of social services, communication and industries. Dar-es-Salaam has a total population of 2,495,940 and of these 1,236,863 are females [23]. The indigenous people in the city are from the Wazaramo ethnic group; and their main activities are subsistence farming, fishing and petty business. Dar-es-Salaam like Arusha is receiving high numbers of migrants annually, coming mainly from other regions in Tanzania in search of better life and other business opportunities. Social interaction is pretty high and this explains the high prevalence of HIV/AIDS (18.8%) [22].

Dar-es-Salaam city and Arusha municipality are served by private and public health services. Public health services where many people go for treatment do not have adequate health facilities and drugs for the patients they are supposed to serve. The only option for many people is to go to traditional healthcare practitioners for various treatments including HIV/AIDS. Even though there is a Traditional and Alternative Medicine Act in Tanzania which allows traditional healthcare practitioners to practice [24] there is no provision for formal referral between modern and traditional healthcare practitioners.

Since the Institute did not know healers who were attempting to manage HIV/AIDS symptoms, the research team began with a consultative meeting with regional medical officers and leaders of traditional healers' associations in order to identify traditional healers that could be partners of collaboration. These were identified as people who were managing HIV/AIDS opportunistic infection in their respective areas. In the two study areas several consultative meetings were held before the gates could be opened to see traditional healers who were managing HIV/AIDS patients. Sometimes a break was called for recollection and consultation in order to proceed with the meeting. The research team used these consultative meetings as a way of cultivating trust and respect with traditional healers. When doors were opened the research team visited the traditional healers who were identified by either regional medical officials or traditional healers' associations.

A detailed open ended questionnaire was administered to each identified traditional healer providing healthcare to HIV/AIDS patients by the research team in a face to face interview. The questionnaire focused on four main areas; background information of a traditional healer, knowledge of HIV/AIDS, type of herbal remedy preparation procedures and rituals, remedies if any for arresting opportunistic infection, the willingness to collaborate in planned research and what they hoped to achieve by collaboration. Whereas, the criteria used to select traditional healers for participation in the project were based on: an average of more than five patients per day and some of them having HIV/AIDS related diseases, a score of more than one of the major clinical symptoms of HIV/AIDS as shown by WHO [3]; and a willingness to disclose medicinal plants used to treat HIV/AIDS and other ingredients.

The questionnaires were completed during individual traditional healers' interview conducted by members of the research team. Additional data was documented in a notebook for detailed documentation and analysis. From the collected data, traditional healers who could participate in a traditional medicine HIV/AIDS project were selected. The selected traditional healers were registered to the ITM record book including addresses and telephone numbers as a way of communicating when the need arose. There was a one-day educational seminar for the selected traditional healers on how to run the project.

The field collected data and those raised during the educational seminar were screened and then transcribed from Kiswahili to English. The data collected were summarized and codes were identified for grouping the information according to issues raised in the introduction. The information from the questionnaires was quantified and put in tables to support the discussion. The information was grouped by code, re-summarized to create a final report of results. The summary of the results is presented below.

## Results

### Entry point of traditional healers and characteristics of traditional healers

Three traditional healers' associations namely Tanzania Traditional Healthcare Practitioners Association (TATHEPA) in Arusha and Dar-es-Salaam Municipalities, African traditional medicine men (ATME) – in Dar-es-Salaam and Chama cha Utafititi wa Magonjwa Sugu (literally meaning an association that investigates chronic diseases/illnesses) Tanzania (CHAUMUTA) in Dar-es-Salaam were involved in the consultative meetings as well as in the project. In both Dar-es-Salaam and Arusha consultative meetings took two days in each association. The key concerns and issues arising during consultative meetings by leaders of traditional healers (chairman, secretary and executive committee) included: what the benefit of the project to traditional healers would be; which referral system would be used, disclosure agreement and patent rights; terms of reference of the collaboration and in contracts; and uncontrolled massive exploitation of medicinal plants by biomedicine prospectors.

In Arusha, the leaders of TATHEPA were in the fore front of uniting traditional healthcare practitioners by making them agree to participate in the study as well as in the collaboration for the benefit of both the traditional healers and the Institute of traditional medicine. Further they were involved in mobilizing traditional healers to come out and be interviewed. A similar response of harmonising traditional healers to appear for interview was noted in Dar-es-Salaam.

In Arusha, with the help of leaders of traditional healers' associations, a total of 132 traditional healers were interviewed; and of these 30.3% (40) were females. In Dar-es-Salaam, on the otherhand, a total of 60 traditional healers were interviewed and of these 25% (15) were females. In all study areas the ages of the respondents were between 25 years and 60 years. Female traditional healers tended to cluster between age 31 to 50 years, whereas male traditional healers tended to form a more or less uniform age distribution (See Table 1 and 2). The analysis of the findings show that most of them had primary education (70%), few had secondary education (5%) and the rest were illiterate. Further, in the study areas nearly all ethnic groups of Tanzania were represented; and hence different cultural backgrounds of the country were captured.

**Table 1:** Distribution of Traditional Healers by Age and Sex in Arusha

Age Distribution	Sex		Total
	male	female	

>20	-	1 (0.8%)	1 (0.8%)
21–30	9 (6.8%)	7 (5.3%)	16(12.1%)
31–40	23 (17.5%)	16 (12.1)	39 (29.6%)
41–50	25 (18.9%)	10(7.6%)	35 (26.5%)
51–60	21 (15.9%)	2 (1.5%)	23 (17.4%)
61–70	11 (8.3%)	4 (3%)	15 (11.3%)
< 70	3 (2.3%)	-	3 (2.3%)
Total	92 (69.7%)	40 (30.3%)	132 (100%)

**Table 2:** Distribution of Traditional healers by age group and sex in Dar-es-Salaam

Age group (years)	male	female	Total
< 20	-	1(1.7%)	1(1.7%)
21–30	3 (5%)	1 (1.7%	4 (6.7%)
31–40	11(18.3%)	6 (10%)	17(28.3%)
41–50	10(16.7%)	4(6.6%)	14 (23.3%)
51–60	12 (20%)	1(1.7%)	13 (21.7%)
61–70	6 (10%)	2(3.3%)	8 (13.3%)
> 70	3 (5%)	-	3 (5%)
Total	45 (75%)	15(25%)	60 (100%)

### Knowledge of HIV/AIDS

On the question of awareness of HIV/AIDS, respondents were rated poor if they could not mention any symptoms of HIV/AIDS, those mentioning one to two symptoms were rated fair, whereas those who mentioned more than three were rated good. The analysis of the findings showed that more than 75% of respondents could at least mention one key symptom as defined by WHO [3] like diarrhoea and persistent cough for more than one month, weight loss, general body weaknesses and repeated fevers. Most of the healers were rated average (49%) on the knowledge of HIV/AIDS (see table 3). Further, the analysis showed that 40% of the respondents have attempted to treat HIV/AIDS patients. Nevertheless, there was some misinterpretation in the management of HIV/AIDS. For example, 10% of the total respondents claimed to have cured HIV/AIDS patients. The indicators to the respondents who claimed to have cured AIDS patients were HIV positive patients becoming negative, giving birth to a healthy child, the patients becoming healthy after the remedies and gaining weight.

**Table 3:** Score on Knowledge of HIV/AIDS symptoms by traditional healers

Knowledge Category	Sex		Total
		
	male	female	
Poor	35 (18.2%)	12 (6.2%)	47 (24.4%)
Moderate	67 (34.9%)	27 (14.1%)	94 (49%)
Good	37 (19.3%)	14 (7.3%)	51 (26.6%)
Total	139 (72.4%)	53 (27.6%)	192 (100%)

One of the problems which the research team encountered in this study was traditional healers' unwillingness to mention medicinal plants used in the management of HIV/AIDS. They were willing to mention the number of medicinal plants that composed their remedies. For instance, 50% of the respondents reported that their remedies were composed of more than ten medicinal plants; and about 10% of the respondents reported that their remedies were composed of more than 100 medicinal plants and other ingredients. In addition, there was a problem of keeping records. Only 30% of the respondents had records of the patients they have treated.

### Handling cultural aspects like rituals in healthcare

The research team learned that there were about 10% of the respondents who believed that there was man made HIV/AIDS (caused by witchcraft, sorcery or evil eye or ancestral spirits) which had similar symptoms to actual HIV/AIDS; and it is only the traditional healers who can identify such cases through *ramli *(divination); and were meeting such patients in their daily practice. The 'man-made' HIV/AIDS required rituals in managing the illness. The research team was sceptical on the man made HIV/AIDS because it is not in line with the theory of HIV/AIDS causation in biomedicine. This was one of the dilemmas the research team faced in associating the 'man-made' HIV/AIDS with the actual cause of HIV/AIDS.

### What traditional healers want from the collaboration?

All traditional healers interviewed showed they would like to collaborate with the Institute of Traditional Medicine. This was further confirmed, during the educational seminar on issues raised by traditional healers like how to handle HIV/AIDS patients when treating them, various modes of transmission of HIV/AIDS, hygiene, record keeping. Traditional healers' responses to the educational seminar were very good. Several questions were asked and mainly focused on; CD4 and CD8, Viral load and ESR, question of turning negative after treatment; health and giving birth to a health baby without HIV/AIDS as a sign to have treated HIV/AIDS and issues of mother to child transmission.

Also issues of depletion of medicinal plants through biopiracy, fires and poor harvesting methods, particularly the rare and endangered species were raised. On the other hand the staff from the Institute of Traditional medicine and some of the medical doctors who participated in the project were also willing to collaborate with the traditional healers and become partners in a planned research project.

## Discussion

Findings on the experience of initiating collaboration with traditional healers have been presented and analysed. Experience has shown that the conceptual appropriateness of methods in initiating collaboration with traditional healers is critical to building a sustainable collaboration [5]. The entry point strategy used by the research team in this study underscores this argument; and hence a meaningful collaboration between/among partners needs to be planned tactfully.

Experience gained in this study points out clearly that leaders of traditional healers' associations and sometimes influential people are gateways to traditional healers; and thus need to be treated very carefully lest one cannot access genuine traditional healers for collaboration. The frequent break for consultations of leaders of traditional healers' associations during dialogue was a learning experience for each group on how to collaborate in the planned project; and it was during that time that trust was cultivated, confidence was built and respect between partners was gradually being learnt and built. The leaders of traditional healers associations understood the importance of the collaboration; similarly the research team, based on the questions and challenges raised by traditional healers' during the dialogue identified areas of collaboration in the provision of healthcare and possible problems that are likely to occur along with strategies to be used to resolve potential identified problems. The research team could have reached the traditional healers through the government hierarchal structure; however, the issue would be what type of traditional healers could be met by using that structure. Good traditional healthcare practitioners do not appear to government officials [3,4]; patients follow them to their respective '*vilinge'*. It was due to trust, confidence and respect shown in the patient's dialogue that the research team managed to access traditional healers who were well known, respected and acknowledged by traditional health practitioners associations and the community at large.

Furthermore, the entry point strategy used by the research team managed to explore the HIV/AIDS symptoms managed by the traditional healers; suggesting that it is very likely traditional healers are treating HIV/AIDS symptoms knowingly and unknowingly. Similar findings have been reported by Scheinman [24] and Kayombo [4] in Tanga and Njombe districts respectively in Tanzania; and Viall [7] in South Africa. Moreover, the strategy has helped the research team assess traditional healers' knowledge on HIV/AIDS which showed that they have a fair knowledge (above 75% could mention at least one symptom of HIV/AIDS) awareness of HIV/AIDS symptoms as defined by WHO [3]. One of the important point in the present findings and others reviewed is that if cases of illness/disease are being managed by herbal remedies for HIV/AIDS, then the indices of success on healthcare of such patients, for effectiveness of such remedies can be postulated to include increased body weight, physical resolution of signs and symptoms, reduced frequency of diarrhoea, reduced incidence of fatigue and depression, lack of gastro-intestinal disturbances, reduced frequency of skin rashes, absence of respiratory infections and reduced medical visits for hospitalisation such improvements are commonly seen amongst HIV/AIDS patients being managed by traditional healers [18].

However, the question of HIV/AIDS patients turning negative, giving birth to a healthy baby and the patient being healthy as indicators of curing HIV/AIDS observed in this study might be reflecting the low understanding of the traditional healers who are attempting to manage HIV/AIDS in Tanzania and even in other African countries south of the Sahara. The noted observation suggest that healthcare seeking behaviours and local knowledge need to be taken seriously in intervention programmes and to promote health in a variety of contexts. It is imperative, therefore, that more knowledge on HIV/AIDS and especially on prevention and caring for the patients should be imparted. This information should include the nature of the disease, prevention and treatment processes. Traditional healers are living with people in the community [2,4,7]; and hence during the collaboration they should be given correct knowledge of HIV for both prevention and care of the HIV/AIDS patients.

It is acknowledged that culture influences a healthcare system and expected associated health outcomes; and in African traditional healthcare system rituals are part of the treatment and healing process of the patients [16,17]. In this study, for example, the "two types" of HIV/AIDS reported by traditional healthcare practitioners suggest that there are no diseases/illnesses without any cultural dimensions in the explanation of the causes of the disease/illness and even the management process. It is very likely through *ramli *that the healers identify the "two types" of HIV/AIDS which may appear alone, or in combination in some patients. Similar observations were reported by Kayombo [4]. After the rituals the patients are relieved from suffering and hopeful their immunity CD4 and body weight can increase.

It is being acknowledged that a treatment of any symptoms of HIV/AIDS patients is prolonging the life of the patient. Literature reviewed show traditional healers are doing a commendable job in alleviating suffering from HIV/AIDS patients in resource poor countries including Tanzania [1-4]. For instance symptoms like skin infection including herper zoster, diarrhoea, coughing, and fever, to mention a few, are being managed by traditional healers [18]. Hence, a need to initiate sustainable collaboration with traditional healers is eminent now. This collaboration might help to find cures to some of the degenerative diseases which have no cure at present.

Notwithstanding, despite the usefulness of traditional healers some of the western trained people and especially physicians as shown in this study seems to down play traditional healthcare practitioners. This observation might be spreading to other physicians as well [6,13]. This is one of the obstacles likely to occur during the collaboration. In contrast to this attitude, the analysis of the findings suggests that traditional healers were more willing to combine forces with the formal health sector [1,3] to fight against HIV/AIDS. This argument was underscored on issues raised during the educational seminar in the term of reference towards how to operationalise the planned project. The issue raised reflects that traditional healers are interested to know more about HIV/AIDS. Further, they have genuine concerns about problems they are likely to encounter in the near future if depletion of medicinal plants goes unchecked/controlled. The Institute of Traditional Medicine have to meet these demands; and in these ways are fostering the collaboration.

Even though the research team assumed that they have won confidence that led to opening the gates to access genuine traditional healers, the overall analysis of the findings in this study seems to suggest that a meaningful collaboration between traditional healers and biomedical practitioners is a long process to be developed systematically by building on what has now been established, for improvement of healthcare to the community [5]. For example, in this study despite being carried out in an urban setting it took two days for the research team in each association to dialogue with leaders so that the doors could be opened for interviewing traditional healers at their respective '*vilinge*'. Again, even after the leaders' associations' interviewing of traditional healers there was resistance to reveal indigenous knowledge used on the management of HIV/AIDS especially disclosure of herbal plants used in the management of HIV/AIDS. Moreover, traditional healers who reported that the formulations of their remedies were composed of 100 medicinal plants seem to suggest in an indirect way that they were not willing to show their remedies to be investigated. In a way they were trying to overwhelm the research team.

The issue of resistance to reveal the knowledge of medicinal plants used in the management of HIV/AIDS is shown in other studies as well [24]. For example, Viall [7] revealed similar observations in South Africa. During fieldwork it was learned that traditional healers were suspicious especially around indigenous concerns of knowledge being stolen; and hence healers were requested not to tell the researchers all the secrets in healing [7]. It is also underscored by 'Van der Maas' [25] experience where traditional healers refused to reveal the ingredients and formulas of their cures out of fear they might be appropriated. Further, Kayombo [4] at Njombe district took almost a week to be able to communicate with a traditional healer whom he was referred to by the cultural officer in Njombe district.

The experience of humiliation during the colonial rule and immediately after independence in many African countries; like being consider witches, the sources of crimes, quacks and charlatans [26,27] makes the traditional healers have a sceptical eye to researchers; and thus the need to cultivate trust and respect is very important during the initiation of the collaboration and in such ways to build confidence. This argument is underscored by Vaill [7] and King [2] and it may take years to cultivate trust, confidence and respect [4].

The observation noted in this study and those reviewed appears to suggest that initiation of the collaboration with traditional healers is not as easy as reflected in the literature reviewed [1]. Further, it seems to suggest the preconditions cited by King [2] were not enough for a meaningful collaboration between the partners; and in addition needs patience and tolerance on both sides through dialogue, educational workshops and seminars; and in this way cultivating respect, confidence and trust involves a process that may take years [7,2,5]. It is not now known what is the status of studies reported to be successful during the initiation of the collaboration [1]; whether they encountered similar problems the ITM and co-researchers faced or not.

## Conclusion

Experience learned from this study shows that initiation of collaboration with traditional healers is not as easy as seen in the literature. It needs gradual cultivation; and in so doing each one learns from the other in what possible ways they can collaborate. The prerequisites of collaboration as shown by King [2] are mandatory for effective collaboration in order to yield good results. With the growing AIDS epidemic in African nations, it is imperative to initiate collaboration so that western-trained medical practitioners learn to value and respect the contributions of traditional healers and to enlist their help in the prevention of HIV/AIDS. Dialogue between traditional healers and modern medical professionals can help alleviate mistrust, build confidence, provide knowledge, and lead to a coordinated approach for controlling HIV/AIDS and other opportunistic infections [7]. It is being acknowledged that healers are well-respected community leaders and are also accessible healthcare options for people with limited financial resources. Further still they have the ability to reach a wide segment of the population. This call for extending training in HIV/AIDS prevention and healthcare to these traditional healers may enhance their services.
